# The Role of TSLP in Atopic Dermatitis: From Pathogenetic Molecule to Therapeutical Target

**DOI:** 10.1155/2023/7697699

**Published:** 2023-04-15

**Authors:** Jialiang Luo, Zhengyumeng Zhu, Yumeng Zhai, Junxiang Zeng, Lei Li, Di Wang, Fan Deng, Bo Chang, Jia Zhou, Ledong Sun

**Affiliations:** ^1^Department of Dermatology, The Fifth Affiliated Hospital, Southern Medical University, Guangzhou, Guangdong, China; ^2^Department of Immunology, School of Basic Medical Sciences, Southern Medical University, Guangzhou, Guangdong, China; ^3^Department of Medical Laboratory, School of Laboratory Medicine and Biotechnology, Southern Medical University, Guangzhou, Guangdong, China; ^4^Department of Bioinformation, School of Basic Medical Sciences, Southern Medical University, Guangzhou, Guangdong, China; ^5^Department of Dermatology, Dermatology Hospital of Southern Medical University, Southern Medical University, Guangzhou, Guangdong, China

## Abstract

Atopic dermatitis (AD) is a kind of chronic skin disease with inflammatory infiltration, characterized by skin barrier dysfunction, immune response dysregulation, and skin dysbiosis. Thymic stromal lymphopoietin (TSLP) acts as a regulator of immune response, positively associated with AD deterioration. Mainly secreted by keratinocytes, TSLP interacts with multiple immune cells (including dendritic cells, T cells, and mast cells), following induction of Th2-oriented immune response during the pathogenesis of AD. This article primarily focuses on the TSLP biological function, the relationship between TSLP and different cell populations, and the AD treatments targeting TSLP.

## 1. Introduction

Atopic dermatitis (AD) is one of the most frequent chronic and relapsing inflammatory skin diseases, which is featured by intense itching along with various clinical symptoms and signs [1]. Over the past three decades, AD affects up to 20% of the population and children in particular among developed countries [2]. Recent study concerning the prevalence of AD in China found that 30.48% of infants and 12.94% of children under 7 years old suffer from AD [3]. Accompanied by persistent or recurrent scratching and inflammation, patients suffering from AD usually develop skin thickening and lichenification during the long-term disease course. Chronic itching of the skin is a characteristic symptom of AD, disturbing patients' daily routines and life quality [4, 5].

The family history of AD or asthma and seasonal allergies is closely related with increased risk of AD in childhood [6, 7]. Apart from genetic disorders, the development of AD is involved with a series of relevant factors, including immune dysregulation, skin microbiota imbalance, epidermal barrier defect, and environmental stimuli (e.g., ambient allergens and stress) [8, 9]. AD as a type 2 immune response usually results in the inhibition of epidermal differentiation and elevation of skin permeability [10, 11]. Moreover, infiltration of inflammatory cell in AD lesional skin is typically featured by the presence of various T cell subpopulations and type 2 innate lymphoid cells (ILC2), prompting a T helper (Th) type 2-predominant inflammation [12, 13]. Keratinocytes, as the largest quantity of cell in epidermis, are serving a significant role in promoting AD proinflammatory environment [14]. Thus, these cells together secrete abundant chemokines, antimicrobial peptides, and cytokines, such as thymic stromal lymphopoietin (TSLP), interleukin- (IL-) 25, IL-33 that drives the infiltration of both innate immune cells (e.g., ILCs and mast cells), and T cells in AD lesional skin [15].

Emerging evidence has revealed TSLP induces Th2-type immune responses via activation of DCs and mast cells; therefore, TSLP is considered as a key molecular in AD physiopathology [16]. In this review, we focus on the role of TSLP associated with different cells during AD pathogenesis and discuss the potential TSLP-targeting AD treatments.

## 2. Overview of TSLP

TSLP was first found in the supernatants of a murine thymic stromal cell line in 1994 [17] and was identified as an epithelial cell-derived cytokine that supports proliferation and development of immature B cell [18, 19]. TSLP, a member of the 4-helix bundle cytokine family, is a paralog of IL-7, sharing an overlapping, but distinct biological profile [20]. The human TSLP gene is located on chromosome 5q22.1, while the mouse TSLP gene is located on chromosome 18 [21]. TSLP binds to TSLPR (TSLP receptor) heterodimeric receptor complex that consists of the TSLPR and the IL-7 receptor *α*-chain (IL-7R*α*) so as to get involved with biological activities. According to sequence analysis, TSLPR is closely related to the common receptor-*γ* chain (*γ*c) [22]. Interestingly, sole TSLPR has low affinity for TSLP, while TSLPR combined with IL-7R*α* induces high affinity for TSLP and triggers subsequent signaling transduction. Amino acid sequence revealed that the identity of TSLP between murine and human is 43% and TSLPR is 39%, suggesting TSLP and TLPLR are homologous between human and mouse [22, 23].

The biological role of TSLP-TSLPR axis is rather conserved in human and mice. On the one hand, TSLP is similar to IL-7 that not only activates the same transcription factor, signal transducer, and activator of transcription 5 (STAT5) but also induces common genes expression. On the other hand, the mechanisms in STAT5 activation are distinctly different between TSLP and IL-7 [24]. Isaksen et al. applied overexpression of kinase-deficient Jak1 in HepG2 which proved that Jak1 is necessary for IL-7-mediated signaling, but not for TSLP-mediated signaling [25].

TSLP is abundantly expressed in the skin, intestine, thymus, lung, and tonsils. TSLP is found in epithelial cells, stromal cells, dendritic cells, and mast cells [26, 27] but is hardly found in hematopoietic cell types and not in endothelial cells [28]. Confrontation with viruses, bacteria, parasites, or TLR (Toll-like receptors) agonists can potently elevate TSLP expression [29–31], but the underlying regulation mechanism remains unknown. Previous study has provided the first evidence of pathogenic stimulation in TSLP induction that human primary small airway epithelial cells remarkedly release TSLP after stimulation of peptidoglycan (a component of Gram-positive bacteria wall) [32]. Additionally, rhinovirus, as one of the most common respiratory viruses, was found to stimulate TLR3 and subsequently elevate TSLP expression in airway epithelial cells [30]. After exposure to proinflammatory cytokines (IL-1*β* and TNF-*α*) and TLR ligands (TLR2, TLR8, and TLR9), gene expression of TLSP is increased in both human and mouse airway epithelial cells [31]. The expression of TSLP is significantly induced by Th2 cytokines (e.g., IL-4 and IL-13) and TLR3 ligand via NF-*κ*B and interferon regulatory factor 3 (IRF3) signaling through TLR3 in human airway epithelial cells [30]. Apart from epithelial cells, TSLP expression is upregulated by IL-1*β* and TNF-*α* in human airway smooth muscle cells [33]. TNF-*α*, TLR3, and TLR4 ligands also increased TSLP in synovial fibroblasts isolated from rheumatoid arthritis and osteoarthritis patients [34, 35]. Allakhverdi et al. reported that TSLP expression is boosted in epithelial cells after damage or trauma [32]. What is more, both *in vivo* and *in vitro* experiments demonstrated that notch-deficient skin keratinocytes lead to persistent defect in skin differentiation/barrier formation which subsequently release high levels of TSLP into circulation system [36]. Of note, poly I:C (a TLR-3 ligand)-activated human keratinocytes decrease the release of TSLP after interferon-*γ* (IFN-*γ*), transforming growth factor-*β* (TGF-*β*), and IL-17 treatment [37].

## 3. TSLP Role during AD Pathogenesis

TSLP has become as an important factor positively correlated with AD. Serum TSLP levels in adults and children with AD are significantly higher compared to those in healthy people [38, 39]. In addition, rising studies have demonstrated that polymorphisms of the TSLP gene are closely associated with the enhanced risk of AD development and progression. According to a study concerning variants in TSLP and its receptors, a significant association was observed between five TSLP single-nucleotide polymorphisms (SNPs) and AD, among which three is found in European American populations (rs1898671, rs11466749, and rs2416259) and two is found in African American populations (rs10043985 and rs2289276). Besides, Gao et al. also found that several IL7R-SNPs and TSLPR-SNPs are closely related with AD [40].

### 3.1. TSLP and Keratinocytes

Keratinocytes are the most abundant cells located in epidermis, serving a significant role in promoting AD [41]. TSLP is highly expressed by keratinocytes in skin lesions of both acute and chronic AD patients. However, TSLP is not found in nonlesional skin in AD patients and lesions of nickel-induced allergy contact dermatitis or cutaneous lupus erythematosus patients [42]. *In vitro* experiments demonstrated that the TSLP expression is significantly elevated in TNF-*α*-inflamed HaCaT [43] and ploy (I:C)-induced NHEK cells [44]. Recent studies hinted that keratinocyte-expressed TSLP is upregulated via TLR [45] and histamine 4 receptor [46] while negatively regulated through glucocorticoid receptor [47] and aryl hydrocarbon receptor [48]. Furthermore, Yoo et al. engineered a kind of transgenic mice (K5-TLSP transgenic mice) that overexpress tetracycline-inducible TSLP in keratinocytes under the control of the keratin 5 promotor [49]. Consistent with the previous *in vitro* findings, skin-specific overexpression of TSLP spontaneously leads to an AD-like phenotype, accompanied with an increase of inflammatory cell infiltration in eczematous lesions, elevated frequency of CD4^+^ Th2-expressing cutaneous homing receptors, and higher levels of IgE in serum in comparison with WT littermates [50]. Their findings indicated that overexpression of TSLP in epithelial cells likely stands for as an early event in allergic inflammation. Of note, TSLP expression in epidermal keratinocytes is necessary for the development of AD-like dermatitis induced by topical application of active vitamin D3 analogue (MC903, an analogue of vitamin D to induce AD-like lesions) [51, 52]. Li et al. unequivocally demonstrated that after the ablation of TSLP in keratinocytes, mice showed neither skin lesions nor infiltration cells and inflammatory cytokines in comparison to WT littermates challenged with MC903 [52].

### 3.2. TSLP and Dendritic Cells (DCs)

It is well known that DCs play a pivotal role in the immunologic cascade, prompting the AD initiation and development. Allergens commonly stimulate DCs through the binding of Fc*ε*RI via specific IgE, subsequently driving Th2 polarization along with dominant secretion of IL-4, IL-5, and IL-13 during AD acute phase [53]. It is reported that TSLP participated in Langerhans cell (LC) migration and activation, implying TSLP as an activator of DC-oriented allergic inflammation in the early stage [54]. Even though DCs are thought to be closely related with AD, direct evidence demonstrating the exact role of DCs in TSLP-induced inflammatory skin remained to be confirmed in the future. Previous study has found that TSLP positively contributes to the maturation of CD11c^+^ DCs and the production of CC chemokines (e.g., thymus and activation-regulated chemokine and macrophage-derived chemokine) by CD11c^+^ subpopulation of DCs to attract Th2 cells [55]. Elentner et al. demonstrated that neither AD-like inflammation was developed nor the level of IgE in serum was upregulated in epidermal LC-depleted mice treated with MC903 in comparison to C57BL/6 mice treated with MC903. Accordingly, they found that the expression of maturation markers by LCs is evidently increased in mice treated with MC903 or in K14-TSLP transgenic mice [56]. It is worthwhile to mention that peroxisome proliferator-activated receptor-*γ* (PPAR*γ*) apparently serves as an important downregulating factor against TSLP-initiated immune responses through inhibiting DC functions and TSLP production in NC/Tnd mice [57]. Furthermore, TSLP is inclined to upregulate the expression of OX40 ligand on DC cell membrane which interacts with OX40 expressed on activated naive T cells to subsequently induce the elevated expression of IL-4 and IL-13 while decreased expression of IFN-*γ* from T cells [50, 58, 59]. TSLP-stimulated DCs also orchestrate the homeostatic expansion of allergen-specific Th2 memory cells as well as prompt the Th2 phenotype polarization via interaction with OX40L [60, 61], thus contributing to the maintenance of chronic allergy-related inflammation.

### 3.3. TSLP and T Cells

*In vitro* experiments have exhibited that mouse TSLP serves as a potent cytokine to support T cell growth. The association between TSLP and T cell lymphopoiesis was further supported by the evidence that *γ*c/TSLPR double-deficient mice developed significantly severe hindrance concerning T cell development compared with *γ*c single-deficient mice [62]. In line with this finding, transgenic mice with overexpressed TSLP recover T cell normal development in IL-7^−/−^ mice [63]. However, it should be noted that both the T cell development and T cell populations' distribution display normal status in TSLPR^−/−^ mice, implying T cell development not strictly dependent on the TSLP-TSLPR pathway under normal conditions [64].

Previous studies have uncovered the biological function of TSLP to induce Th2 differentiation due to the direct interaction on CD4^+^ T cells in mice. Omori and Ziegler found that TSLP preferentially contributes to Th2 cell differentiation from naïve CD4^+^ T cells due to the induction of IL-4 expression in CD4^+^ T cell without the presence of the addition of IL-4 and antigen presenting cells. Thus, the blocking of IL-4 suppresses TSLP-induced Th2 polarization [50]. Additionally, TSLP directly acts on antigen-stimulated CD4^+^ T cells and promotes the upregulation of Th2 cytokine. It is evidenced by the observation that human TSLP not only contributes to the activation and differentiation of CD8^+^ T cells into cytotoxic T cells but also acts as a survival factor for CD8^+^ T cells [65]. What is more, TSLP can also directly interact with TSLPR expressed on TCR-activated human and murine CD8^+^ T cells, thus boosting CD8^+^ T cell survival and inducing the expression of antiapoptotic protein, Bcl-2, while hardly exerting function on CD8^+^ T cell homeostatic proliferation [66].

### 3.4. TSLP and Other Immune Cell Populations

Beside from the discussion of TSLP with keratinocytes, DCs, and T cells, TSLP is also closely associated with other immune cell populations. A growing body of evidence has demonstrated that skin inflammatory infiltration cells, including mast cells and eosinophils, are remarkedly increased accompanied with skin epidermal thickening in the K5-TSLP transgenic mice with TCR*β*^−/−^ background [49]. *In vitro* experiments found that the proinflammatory Th2 cytokines, including IL-5, IL-6, and IL-13 produced and secreted by human mast cells, are attributed to the synergistical stimulation of TSLP, IL-1*β*, and TNF-*α* [32]. What is more, the addition of TSLP with bone marrow progenitor cells for 4 weeks supports mast cell proliferation and differentiation [67]. Recent study elucidated that TSLP promotes the degranulation of skin mast cells which is significantly dependent on STAT5 and contributed by JNK activity [68]. In turn, mast cells are capable of regulating the epithelial TSLP expression via Fc*ε*RI during allergic rhinitis development [69].

The elevation of TSLP expression mediates eosinophils, DCs, and mast cells in MC903-induced RAG^−/−^ mice. Single TSLP treatment can increase the migration of eosinophil, and the combination of TSLP with other cytokines can further enhance eosinophil migration via the phosphorylation of L-plastin [70].

TSLP has been found to promote basophil hematopoiesis, when cultured with bone marrow progenitor cells for 5 days. TSLP is capable of maturing a functionally distinct basophil subgroup without strictly requiring IgE. In addition, TSLP-induced basophils would release IL-4, thus triggering the Th2 response and accelerating AD progression [71, 72].

ILC2s, as one of the ILC subsets, are distributed in healthy human skin and found abundantly enriched in lesional skin of people suffering with AD [73]. In AD murine model induced by MC903, ILC2s play a significant role in the skin inflammation development [74]. A recent study has discovered that TSLP and IL-33 reciprocally promote each other's protein expression, thereby further enhancing ILC2 activation and expansion during innate allergic airway inflammation [75].

## 4. Targeting TSLP Treatment in AD Patients

As described, accumulating evidence indicated that TSLP is a key molecule in the pathogenesis of AD. Released by epidermal keratinocytes due to a series of stimulation, TSLP promotes Th2 responses as well as the pathway for pruritus via interacting with a subpopulation of sensory neurons. Thus, TSLP is a promising therapeutic target for ameliorating or preventing AD progression. Tezepelumab (AMG-157/MEDI9929) is an IgG2*λ* human monoclonal antibody with the aim at circulating TSLP. In terms of mechanism, tezepelumab binds to the TSLP receptor, which therefore interferes the interaction of TSLP with its receptor and ultimately blocks the downstream inflammatory pathways. Tezepelumab has conducted the phase 1 clinical trial (NCT00757042) and phase 2a (NCT02525094) randomized, double-blinded, and placebo-controlled studies in AD patients.

In the phase 2a trial, 113 patients with moderate to severe AD were randomized 1 : 1 at first, following either subcutaneous tezepelumab (280 mg) combined with class 3 topical corticosteroids (TCS) treatment or a placebo combined with TCS treatment every 2 weeks [76]. At week 12, the percentage of EASI-50 (a 50% decrease in the Eczema Area and Severity Index) was evaluated, and the results showed that patients in tezepelumab combined with TCS group exhibited a greater percentage of EASI-50 compared to the placebo combined with TCS group (64.7% vs. 48.2%; *p* = 0.091). Moreover, multiple improvements were observed among patients at clinical endpoint, and biomarker-defined subgroups displayed greater efficacy of tezepelumab, indicating the potential benefits of TSLP blocking in treating AD patients. Nevertheless, no statistically significant improvements were achieved, including EASI50 response, 1EASI75 response, and pruritus numeric rating scale. Apart from efficacy verification, researchers also evaluated the safety of tezepelumab that the occurrence of treatment-emergent adverse events (TEAEs) was similar between two treatment groups. This clinical trial explored a potential agent to ameliorate AD, but with limited samples. In the light of the critical role of TSLP in AD pathogenesis in the previous preclinical studies, more clinical studies with tezepelumab application are required to confirm these findings in the phase 2a trial. Besides, a phase 2b trial of tezepelumab enlisted AD patients to further testify the safety and effectiveness of tezepelumab as monotherapy and adjunct therapy in combination with TCS (NCT03809663) [77]. It is worthwhile to mention that another TSLP-targeting drug, MK-8226, was conducted in a phase 1b trial (NCT01732510) but was hauled on the halfway of the trial without statistic recorded [73]. Beside from clinical trial in treating AD, tezepelumab has completed phase 2 clinical trial in patients with asthma (NCT02054130) and has two ongoing phase 3 trials in patients with uncontrolled, severe asthma [78]. What is more, CSJ 117 which is an anti-TSLP monoclonal antibody fragment (Fab) has completed a 3-month phase 1 trial (NCT03138811) among patients suffering with mild asthma [73]. In conclusion, although there is no clinical trial supporting the efficacy of TSLP-targeting drugs against AD at present, new agents targeting TSLP or TSLPR in the future are still expected highly.

## 5. Conclusion and Prospect

**Figure 1:**
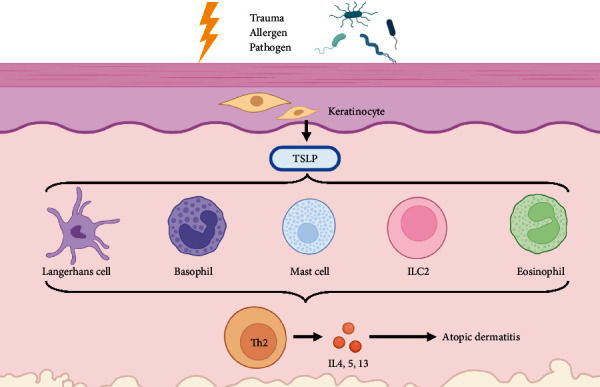
The schematic of TSLP in AD pathogenesis. Firstly, the release of TSLP from keratinocytes is elevated after exposed to allergens, pathogens, and trauma. TSLP directly acts on multiple immune cells (including LCs, basophils, mast cells, eosinophils, and ILC2s) to initiate and maintain allergic inflammation. Then, these immune cells synergistically drive the Th2-predominating immune responses to promote atopic dermatitis.

The association between TSLP and AD has been confirmed by rising evidence. The SNPs of TSLP gene are positively correlated with the high occurrence of AD among people. The current available evidence proves that the overexpression of TSLP in keratinocytes deteriorates AD-like lesions in mice, while ablation of AD impedes the development of AD. As TSLP is involved with numerous cell populations during AD (Figure 1), TSLP not only promotes Th2 response through mediating DC-Th2 axis during the induction phase but also elicits activated T cells and the secretion of Th2 cytokines during the effector phase. Further investigations on TSLP will have a better understanding of its biological function on AD and related allergic diseases. To date, the common therapy against AD in clinic consists of moisturization, topical and systemic application of corticosteroids, and application of calcineurin inhibitors. Since numerous basic studies have suggested TSLP is a promising therapeutic target against AD and other allergic diseases, further clinical trials are required to validate whether TSLP-targeting therapies could prevent the progression of AD, which is expected to improve AD patients' quality of daily life and alleviate AD-related allergic disorders.

## Data Availability

No original data were used in this study.
